# Frequency of *KRAS* p.Gly12Cys Mutation in Brazilian Patients With Lung Cancer

**DOI:** 10.1200/GO.20.00615

**Published:** 2021-05-06

**Authors:** Rodrigo Cavagna, Flávia Escremim de Paula, Débora Sant'Anna, Iara Santana, Vinicius D. da Silva, Eduardo C. A. da Silva, Carlos E. Bacchi, José E. Miziara, Josiane M. Dias, Pedro De Marchi, Leticia F. Leal, Rui M. Reis

**Affiliations:** ^1^ Molecular Oncology Research Center, Barretos Cancer Hospital, Barretos, Brazil; ^2^ Molecular Diagnostic Laboratory, Barretos Cancer Hospital, Barretos, Brazil; ^3^ Department of Pathology, Barretos Cancer Hospital, Barretos, Brazil; ^4^ Bacchi Laboratory, Botucatu, Brazil; ^5^ Department of Thoracic Surgery, Barretos Cancer Hospital, Barretos, Brazil; ^6^ Department of Medical Oncology, Barretos Cancer Hospital, Barretos, Brazil; ^7^ Oncoclinicas, Rio de Janeiro, Brazil; ^8^ Barretos School of Health Sciences, Dr Paulo Prata—FACISB, Barretos, Brazil; ^9^ Life and Health Sciences Research Institute (ICVS), School of Medicine, University of Minho, Braga, Portugal; ^10^ ICVS/3B's—PT Government Associate Laboratory, Braga/Guimarães, Portugal

## INTRODUCTION

Lung cancer is the deadliest cancer worldwide, and in Brazil.^1,2^ In the past decade, targeted therapies have revolutionized the clinical management of lung cancer, particularly in non–small-cell lung cancer (NSCLC) subtype.^3-8^ The most successful examples of targeted therapies are the EGFR and ALK inhibitors, used for *EGFR*-mutated and *ALK-*translocated tumors, respectively.^9,10^

*KRAS* is one of the most frequently mutated genes in NSCLC. The frequency of *KRAS* mutations varies among distinct populations, accounting for approximately 25% in Whites and < 10% in East Asians.^11^ *KRAS* driver mutations are mostly located in codons 12 and 13, and the most frequent one is the p.Gly12Cys (c.34G>T) mutation.^12-18^ In lung cancer, *KRAS* mutations are associated with smokers and with a more aggressive phenotype.^12,19-22^ Efforts have been made in the past decade for rendering *KRAS* mutations susceptible to targeting.^23^ However, until lately, *KRAS*-mutated tumors were, unfortunately, undruggable.^10^

Recently, the agents AMG-510 (sotorasib, Amgen, Thousand Oaks, CA) and MRTX849 (adagrasib, Mirati Therapeutics, San Diego, CA) were developed to target the *KRAS* p.Gly12Cys mutation.^24,25^ These specific inhibitors locked *KRAS* p.Gly12Cys mutation in an inactive state, hampering the oncogenic signals and allowed the normal function of remained wild-type *KRAS*.^24-26^ In a phase I study, 32.2% (19 out of 59) of sotorasib-treated patients presented with objective response, and 88.1% (52 out of 59) presented with the disease control.^26^ In a phase I and II study, 94% (17 out of 18) adagrasib-treated lung patients presented with disease control, and objective response was not yet available (KRYSTAL-1 study; ClinicalTrials.gov identifier: NCT03785249).^25^

The frequency of *KRAS* p.Gly12Cys in admixture NSCLC populations remains scarce. Herein, we report the frequency of the *KRAS* p.Gly12Cys mutation in a series of 844 Brazilian NSCLC cases, followed by the data gathered from Brazil's previously reported studies.

## METHODS

This retrospective study included 844 patients diagnosed with NSCLC. Seven hundred fifty-four patients were diagnosed at Barretos Cancer Hospital (BCH), and 90 patients were diagnosed at Bacchi Laboratory. Tobacco exposure, performance status, and overall survival data were provided for a subset of patients (BCH). This study was approved by the local IRB (Project no. 630/2012), and all procedures were performed following the Helsinki Declaration.

*KRAS* mutational status was evaluated from FFPE tumor tissue using different methodologies. The cases diagnosed at Barretos Cancer Hospital from 2014 to 2017 (n = 319) were genotyped by polymerase chain reaction followed by direct Sanger sequencing, and from 2018 to 2020 (n = 435) was assessed by next-generation sequencing, using the TruSight Tumor 15 (Illumina Waltham, MA) as reported by our group.^12,27,28^ The cases diagnosed at Bacchi Laboratory were analyzed by qPCR TaqMan-MGB allelic discrimination assay (n = 67) and by FoundationOne (n = 23) between 2018 and 2020.^29,30^ Genetic ancestry was analyzed in a subset of patients from Barretos Cancer Hospital (n = 660 out of 844), as previously described.^12^

For statistical analysis, the percentage was used to describe categorical variables, and medians were used to describe continuous variables. Fisher’s exact test and χ^2^ test were used for the association between *KRAS* mutations and the clinicopathogic data. The log-rank test and the Kaplan-Meier curves were used to analyze patients’ overall survival. The Cox regression method was used to investigate the association of clinicopathologic data to the outcome (death). All tests were made in the software IBM SPSS Statistics version 22 with a limit of statistical significance of 0.05.

## RESULTS

We evaluated the frequency of *KRAS* mutations in a series of 844 NSCLC (Table 1). The median age of the cohort was 64 years, 55.4% (n = 468 out of 844) were male, 88.2% (n = 744 out of 844) were adenocarcinoma, and 2.2% (n = 19 out of 844) were squamous-cell carcinoma. Concerning tobacco consumption, 63.5% (n = 536 out of 844) were current or quitter smoking, 65.3% (n = 552 out of 754) were diagnosed in an advanced stage of the disease, and 9.7% (n = 82 out of 844) were diagnosed with worse Eastern Cooperative Oncology Group performance status (ECOG PS; Table 1).

**TABLE 1:**
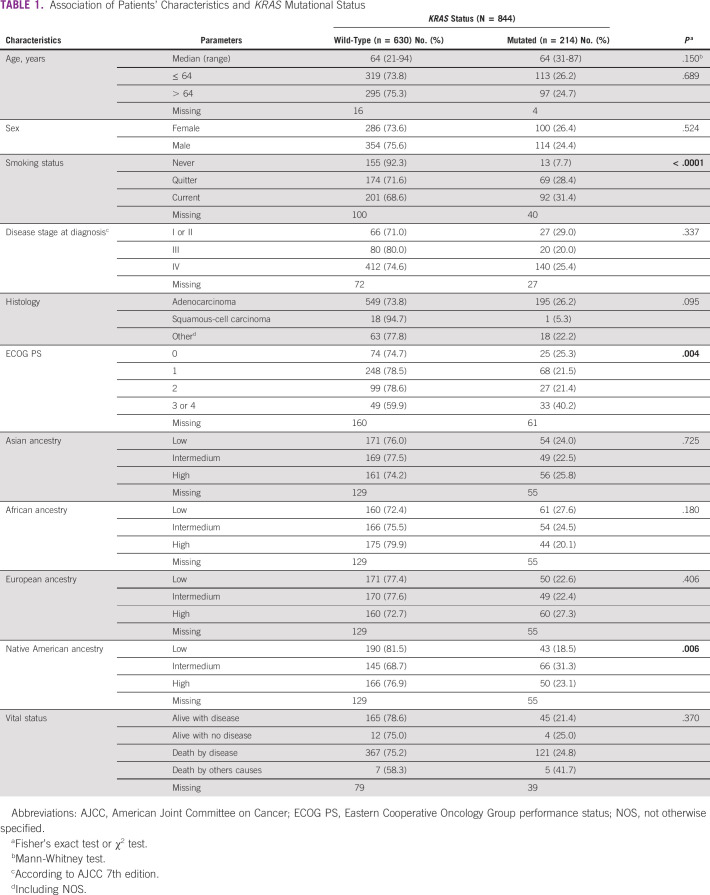
Association of Patients' Characteristics and *KRAS* Mutational Status

*KRAS* was mutated in 214 cases (25.3%; Table 1). A detailed description of KRAS mutation variants is described at Appendix Table A1. Briefly, in the adenocarcinoma subtype, 26.2% (n = 195 out of 744) were *KRAS*-mutated, with p.Gly12Cys being the most frequent mutation identified in 9.4% (n = 70 out of 744), followed by p.Gly12Val in 6.2% (n = 46 out of 744). Among squamous-cell carcinomas, 5.3% (n = 1 out of 19) were *KRAS*-mutated (p.Gly12Asp). Concerning other histologies, 22.2% (n = 18 out of 81) were *KRAS*-mutated, with p.Gly12Cys being the most frequent mutation identified in 7.4% (n = 6 out of 81; Appendix Table A1).

**FIG 1:**
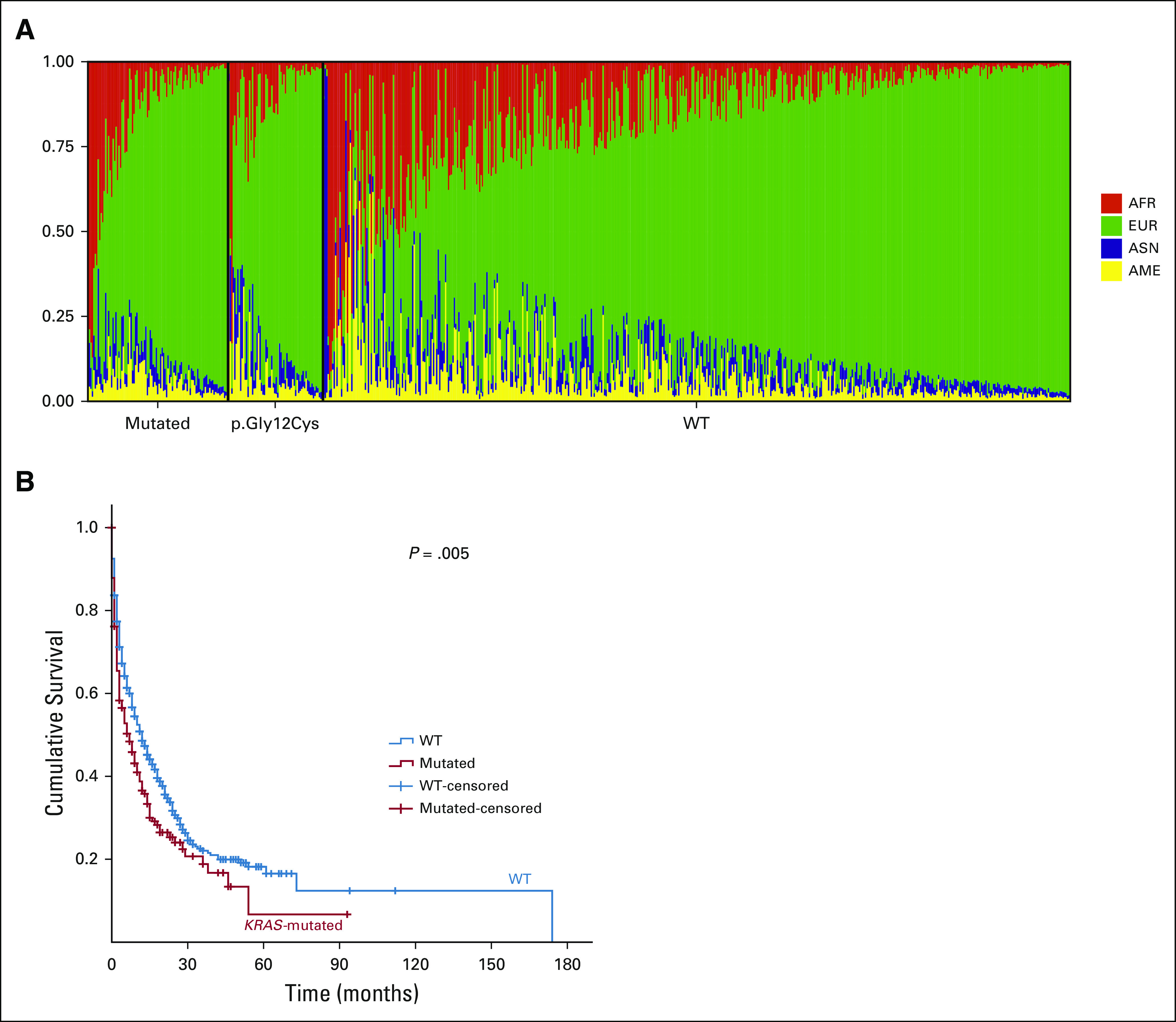
(A) Ancestry background of patients, divided in mutated patients and wild-type patients (n = 660). (B) Kaplan-Meier comparing wild-type patients with mutated patients. AFR, African; AME, Native American; ASN, Asian; EUR, European; WT, wild-type.

The genetic ancestry evaluation in a subset of patients (n = 660 out of 844) showed the following proportion of ancestry background: 72.2% for European, 14.0% for African, 6.4% for Asian, and 7.5% for Native American (Fig 1A).

*KRAS* mutation status was further associated with clinicopathologic and ancestry features (Table 1). Significant associations were found between the presence of *KRAS* mutations and smoking status, ECOG PS at diagnosis, and Native American ancestry (Table 1). Patients harboring *KRAS* mutation had worse overall survival than wild-type patients (Fig 1B). Besides, smoking and higher ECOG PS at diagnosis were significantly associated with higher risk of death by multivariate Cox regression analysis (*P* < .0001 and *P* < .0001, respectively).

**FIG 2:**
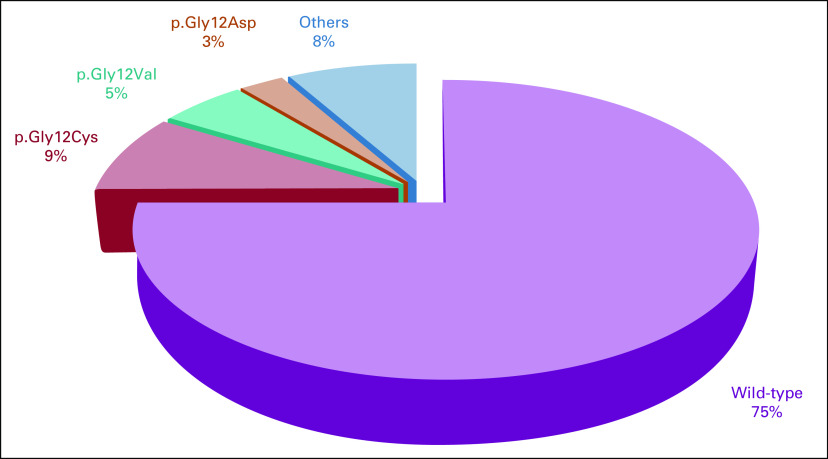
Frequency of *KRAS* mutations in Brazilian patients with non–small-cell lung cancer (n = 3,247).

We further gathered *KRAS* mutational status reported in the NSCLC Brazilian population (Table 2; Fig 2). Among the 3,247 cases, the *KRAS* mutational frequency was 25.0% (n = 813 out of 3,247)—ranging from 15% to 31% among studies (Table 2). The *KRAS* p.Gly12Cys mutation frequency was 35.0% (n = 285 out of 813) of the *KRAS*-mutated cases, corresponding to 9% (285 out of 3,247) of all Brazilian NSCLC cases—ranging from 6.0% to 12.0% (Table 2; Fig 2).

**TABLE 2:**
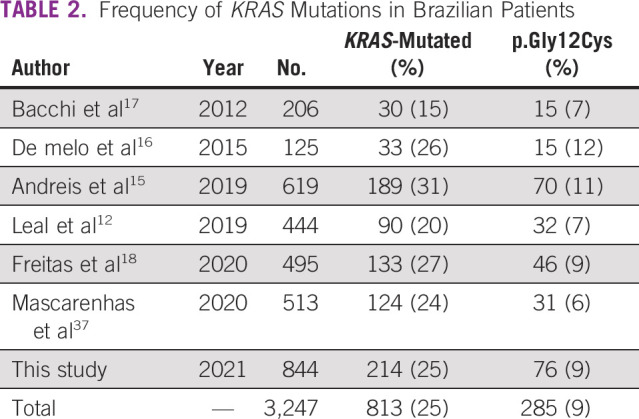
Frequency of *KRAS* Mutations in Brazilian Patients

## DISCUSSION

The *KRAS* p.Gly12Cys mutation became a new target for personalized therapy with the sotorasib and adagrasib.^23,25,26,31^ Our study analyzed the frequency of p.Gly12Cys mutation in the Brazilian NSCLC population. We observed that 25% of the 3,247 cases were *KRAS*-mutated, and the most common variant was the p.Gly12Cys, present in 285 (9%) of the cases. Currently, expanded access is available for Brazilian patients and also for patients around the world, since both are non–US Food and Drug Administration-approved drugs. Once US Food and Drug Administration approves any of these drugs—sotorasib and adagrasib—compassionate drug use may be the option for obtaining access for Brazilian patients.

In our study, the presence of *KRAS* mutations was associated with smoking status (current or quitter) and worse overall survival. These data are in agreement with the literature.^12,19-22^ A recent review reported that *KRAS* mutations are present in 18%-32% of lung adenocarcinoma, 12.8% of large cell carcinoma, 10% of adenosquamous carcinomas, and 1.6%-7.1% of squamous-cell carcinomas in White patients.^32^ Moreover, African-American patients with NSCLC are more frequently identified with *KRAS* mutations than White patients.^32^ The frequency of *KRAS* mutations in Western populations with lung adenocarcinoma is about 26% and about 6% in the squamous-cell carcinoma population.^33^ In Asian patients, the frequency of *KRAS* mutations is 11.2% of patients with NSCLC.^33^ According to The Cancer Genome Atlas, *KRAS* mutations are found in 33% of lung adenocarcinoma.^34^ A study involving 5,738 NSCLC cases reported 14% of *KRAS*-mutated cases in Latin American except for Brazil (Argentina, Mexico, Colombia, Peru, Costa Rica, and Panama).^35^

The role of genetic ancestry in *KRAS* mutational status in NSCLC is poorly explored. A recent metadata analysis showed that *KRAS* mutations were more present in White and Black NSCLC patient groups than in Asian.^36^ In a previous study, our group reported that *KRAS* mutations were associated with low Asian genetic ancestry background.^12^ In the current study, using a panel of genetic ancestry markers, these findings were not confirmed in a multivariate analysis. Therefore, further studies using an admixture of populations are needed to clarify this important issue.

In conclusion, we showed that approximately 10% of Brazilian patients with NSCLC harbor the *KRAS* p.Gly12Cys variant and are therefore potentially responsive to the new anti-KRAS agents.

## Figures and Tables

**TABLE A1:**
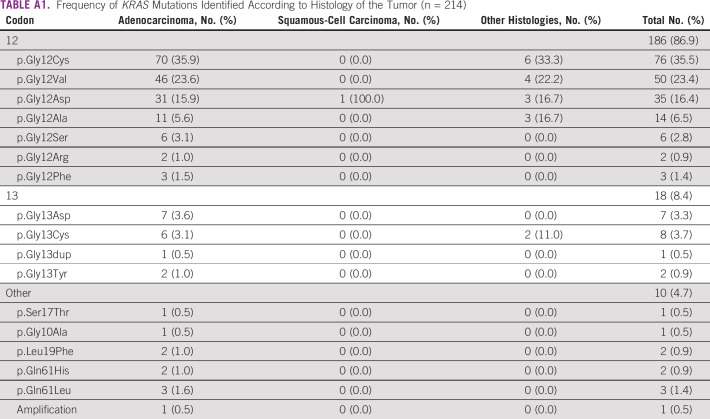
Frequency of *KRAS* Mutations Identified According to Histology of the Tumor (n = 214)
